# The Actor-Dueling-Critic Method for Reinforcement Learning

**DOI:** 10.3390/s19071547

**Published:** 2019-03-30

**Authors:** Menghao Wu, Yanbin Gao, Alexander Jung, Qiang Zhang, Shitong Du

**Affiliations:** 1 College of Automation, Harbin Engineering University, Harbin 150001, China; wumenghao@hrbeu.edu.cn (M.W.); 18846425693@hrbeu.edu.cn (Q.Z.); dushitong@hrbeu.edu.cn (S.D.); 2 Department of Computer Science, Aalto University, 02150 Espoo, Finland; alexander.jung@aalto.fi

**Keywords:** reinforcement learning, continuous control, DDPG, dueling network, advantage

## Abstract

Model-free reinforcement learning is a powerful and efficient machine-learning paradigm which has been generally used in the robotic control domain. In the reinforcement learning setting, the value function method learns policies by maximizing the state-action value (*Q* value), but it suffers from inaccurate *Q* estimation and results in poor performance in a stochastic environment. To mitigate this issue, we present an approach based on the actor-critic framework, and in the critic branch we modify the manner of estimating *Q*-value by introducing the advantage function, such as dueling network, which can estimate the action-advantage value. The action-advantage value is independent of state and environment noise, we use it as a fine-tuning factor to the estimated *Q* value. We refer to this approach as the actor-dueling-critic (ADC) network since the frame is inspired by the dueling network. Furthermore, we redesign the dueling network part in the critic branch to make it adapt to the continuous action space. The method was tested on gym classic control environments and an obstacle avoidance environment, and we design a noise environment to test the training stability. The results indicate the ADC approach is more stable and converges faster than the DDPG method in noise environments.

## 1. Introduction

Autonomous navigation is a core research area of the mobile robot, and the obstacle avoidance technique has been treated as a planning problem [1]. An efficient navigation system requires both global path planning and local motion control ability. The local motion usually uses sensory information to determine a motion that will avoid collision with unknown obstacles [2]. The classical solutions such as simultaneous localization and mapping (SLAM) enable autonomous vehicles to safely roam in unknown environments while incrementally building a map of it [3]. The robot uses laser or cameras as major sensors to scan a great many points in an area, based on this map it can avoid collisions. But avoiding obstacles based on a complex world representation can be inconvenient and lacks flexibility. Besides, some basic obstacle avoidance algorithms determine a suitable distance and are based on recent sensory data to ensure the real-time avoidance. However, to optimize these algorithms, lots of environmental circumstances should be considered and tested [1]. The visual-based navigation system recognizes objects and measures the distance from them by monocular or binocular cameras to avoid obstacles. However, the visual information is easily affected by environmental conditions such as the position of light sources, illumination intensity, etc. [4]. To solve the obstacle avoidance task based on distance measurement, sensors such as lasers and LiDARs can provide simple and effective solutions. The sensors are employed to collect information around the robot, thus the robot can perceive the relative position between itself and the environmental obstacles. Traditional control algorithms are based on expert knowledge and experimental experience to make the sensors and motors coordinate to avoid collisions. It has a high training cost and is not flexible. With the rise of artificial intelligence in recent years, approaches based on deep learning have achieved lots of impressive results [5,6,7,8,9]. Combining with transfer learning technique, the pre-trained local motion planning model and knowledge in the simulator can be effectively used in real robots to achieve obstacle avoidance [10]. There are many excellent works [11] training models in simulators such as gazebo [11] and Mojuco [12]. Typically, robots such as Turtlebot measure distance from environments with onboard LiDAR and sonar to avoid unpredictable obstacles. Based on the sensor input, the robot can finish navigation tasks with a pre-trained model. The model and knowledge can be acquired by means of reinforcement learning. Therefore, the study of reinforcement learning algorithms and approaches to improve training performance has more practical significance to navigation field presently.

Reinforcement learning (RL) is a mathematical framework for autonomous learning optimal control strategy through trial and error in a wide range of fields besides engineering and robotics [13]. The control strategy or policy is a mapping between states and actions, which enables the agent to have knowledge about selecting a good action based upon the current state and its experience during interaction with an environment. This learning process continues until the agent acquires a promising performance and the whole process is fully driven by the reward. For obstacle-avoiding robots, the sensory input can be regarded as states, and the operation of the motor is regarded as the action. Whether the collision occurs or not can be defined as rewards. Through this change, an obstacle avoidance task can be formalized as a standard RL process. Extracting the useful information from the environment (forms such as images, text, and audio) is a key capability for training the agent. With the recent advances in deep learning (DL), relying on the neural networks’ powerful function approximation and representation learning properties allows an RL agent to efficiently learn features and patterns from high-dimensional data with multiple processing layers models [14]. It has dramatically accelerated the developing process of RL, and deep reinforcement learning (DRL) could be applied to more fields and learn knowledge end to end by integration of RL and neural networks. There is a range of successful neural network architectures, such as convolutional neural networks (CNN) [15], multilayer perceptrons, recurrent neural networks [16], generative adversarial nets (GAN) [17], etc., which dramatically improves the state of the art in applications such as object detection, speech recognition, and language understanding [14]. DRL algorithms can deal with decision-making problems that were previously intractable with high-dimensional state input and large action spaces [18], which has made significant progress, and it became easier to train a more complex neural network model than before.

In this paper, we focus on improving the training performance of RL algorithms by providing new training technique and apply it in an obstacle avoidance simulator to discuss the practicability in navigation field. This method combines the benefit from the actor-critic framework and the dueling network architecture [19]. We refer to this hybrid approach as the ADC algorithm. The ADC algorithm operates well in a continuous action space since it has an actor network, which directly optimizes policy and selects actions. On the other hand, the dueling-critic network can estimate state-action value and action-advantage value. By combining the two estimated values in a technique we present, the estimation of Q-value can be insensitive to the change of environmental noise, thereby improving the training stability. The dueling-critic adopts the framework design like dueling network [19], which is an efficient technique that decouples the state and action pairs; therefore, it evaluates the action value independent of states. Our method provides a more accurate estimation of the state-action value at each time step, and it is an important factor for guiding the actor to update its policy network. However, the original dueling network can only work in discrete action space, since it is based on standard the deep Q-learning network and the action advantage is a relative action value in each state, relative to other unselected actions. In the continuous action space, the unselected actions are countless, and it is impossible to evaluate each unselected actions’ advantage value. We introduce the concept of action interval, converting the action’s advantage to action interval’s advantage value, which makes it possible to use this technique in continuous action spaces.

To test our approach’s performance, we apply our method to the gym Pendulum-v0 environment and the obstacle avoidance environment, all tasks are in continuous action space. To explore the stability of the ADC method, we manually add noise to the environments. From the results show, our method operates well in the continuous control tasks and the training process is more efficient than DDPG algorithm [20] especially in the environments with noise input. The contributions of this paper are summarized as follows:

- We provide a novel network structure (ADC) working in continuous action space, which can decouple the states and actions and estimate state value and action advantage separately in an environment.
- We introduce the concept of action interval’s advantage, which makes it possible for the advantage technique to be used in continuous action domain.
- Based on the ADC structure, we propose an algorithm that is effective at learning policies for continuous control tasks. This is fully model-free, and it leads to stable long-term behavior.

The rest of paper is organized as follows. Section 2 gives a brief review of the main RL-related techniques for improving training performance and the robotics applications. In Section 3 we formalize the problem setup and provide some necessary background knowledge of RL. Our main contribution is in Section 4 which discusses our approach which combines dueling network architecture with the actor-critic network and presents the details of the network such as the action interval’s advantage and dueling network’s aggregating module. Section 5 we present the experimental results of our method on a gym classic control simulator and navigation simulator. Some general discussions are presented in Section 6 and Section 7 concludes the paper with some potential use cases.

## 2. Related Work

Many researchers have studied RL algorithms and relevant techniques to improve their training performance. At the same time, many research groups have narrowed the gap between the algorithms and practical applications. In particular, the applications in continuous control have drawn more attention, which has great significance in the field of robotic navigation.

Mnih et al. [21] developed the first standout DRL algorithm, the Deep Q-Network (DQN) algorithm, which develops a novel artificial agent achieved human-level performance in playing Atari series games, directly from raw video input. Silver et al. [22] set a landmark in artificial intelligence (AI) in playing the game of Go based on supervised learning and RL. DRL is poised to revolutionize the field of AI and represents a big step towards building fully autonomous or end-to-end systems with a higher-level understanding of the visual world [18]. Henceforth, a number of new RL algorithms and techniques have sprung up to improve the training performance in their own way. Hasselt et al. [23] presented a double-Q estimator for value-based RL methods to decrease the overestimated *Q* value, hence improve the agent’s training performance. Wang et al. [19] improve the accuracy of *Q* value estimation by adopting two split networks, one for estimating state value and the other one for estimating its action value. In contrast to modifying networks’ structure, Schaul et al. [24] investigated the prioritized experience replay (PER) method to make experience replay more efficient and effective, this prioritization can lead to quickly convergence in the sparse reward environment. Nair et al. [25] introduced massively distributed DRL architecture which consists of parallel actors and learners and it uses a distributed replay memory and neural network to increase the computation. These advances in algorithms drove lots of researchers to do experiments in applications such as visual navigation and robot control. Barron et al. [26] explored virtual 3-D world navigation with deep Q-learning method, the trained agent can have good performance with a shallow neural network. Relative research did by Mirowski et al. [27] they formulated the navigation task as an RL problem and trained an agent to navigate in a complex environment with dynamic elements. Haarnoja et al. explored a series of tasks and methods enable real robots to learn skills [28,29,30], and they presented the soft actor-critic [31] method to improve the sampling efficiency. As for the application of RL in the control field, the first thing we should consider is the action space. Because the majority of previous RL methods are in domains with discrete actions which are based on value function estimation [32]. For the real-world applications related to physical control such as robotics, an important property is the continuous (real-valued) action spaces. The methods based on the value functions such as deep Q-learn cannot be straightforwardly applied to continuous domains since it relies on finding the action that maximizes the action-value function and requires an iterative optimization process at every step [20]. So, the exploration of RL algorithms in continuous action space is important and practical work.

For continuous control tasks, simply discretizing the action spaces and using value-based methods such as DQN is a feasible way. Obviously, it must drop off a lot of information about the action space, thus undermines the ability to find the true optimum policy. Large action spaces are difficult to explore effectively and making the training process intractable with traditional value-based methods. Therefore, lacking most of the action spaces’ information will result in poor performance. Another series of algorithms developed based on policy-gradient methods [33], which directly optimize the parameters of a stochastic policy through local gradient information obtained by interacting with the environment using the current policy [34]. The critical challenge of policy-based methods is finding a proper score function to evaluate how good or bad a policy is. To solve it, actor-critic approaches have grown in popularity in the continuous domain, which take advantage of prior researching experience and they are capable to select actions in a continuous domain with a temporal-difference learning value function. Based on this hybrid framework, Mnih et al. [35] proposed an asynchronous variant of actor-critic (A2C) method which surpasses the origin actor-critic in convergence time and performance. Lillicrap et al. [20] presented the deep deterministic policy-gradient (DDPG) algorithm it robustly solved a variety of challenging problems with continuous action spaces. O’Donoghue et al. [36] gave a similar technique such as DDPG which combines the policy gradient with off-policy Q-learning (PGQL). Among these actor-critic-based methods, the critic network ways of estimating the state-action value are all same. Notably, these estimations serve as a signal to guide the actor network select better actions then update the policy. Therefore, we intend to present a method which has more precise and proper state-action values’ estimation in the critic network, then potentially improve the overall performance.

There is a lot of recent work that solves robotic navigation tasks with RL approaches. Tai et al. [37] trained a mapless motion planner for navigation tasks with an asynchronous DRL method, which can be directly applied in unseen environments. The motion planer was trained end to end based on the sparse laser sensors. Zhu et al. [38] presented an RL-based model for target-driven visual navigation tasks, the model addressed the issues such as lacking generalization capability and data inefficiency. Xie et al. [39] presented a method based on a double-Q network for obstacle avoidance tasks, using monocular RGB vision as input. Zuo et al. [40] built a robotic navigation system based on the Q-learning method, which is useful for robot to quickly adapt unseen environments with sonar measurements input. Zhang et al. [41] proposed a successor-feature-based DRL algorithm which used for obstacle avoidance task rely on raw onboard sensors’ data. Tai et al. [42] presented the deep-network structure to do obstacle avoidance tasks, they tested their model in real-world experiments and showed the robot’s control policy has high similarity with human decisions. Khan et al. [43] proposed a self-supervised policy-gradient algorithm and applied it in a LiDAR-based robot. These works showed the RL methods can make full use of robots’ sensory input, and map the input to the appropriate action output for safely walking without collisions, and the models trained in simulators in these works can be successfully transferred to the robot in the real world for the same tasks.

## 3. The Problem Setup

The RL problem is meant to be a straightforward framing of the problem of learning from interaction with environments E over several discrete time steps to achieve a goal [44]. At each time step *t*, the agent receives a state $s_{t}$ in the environment’s state space S and selects an action, $a_{t}\in A\left(s_{t}\right)$ according to a policy $\pi (a_{t}|s_{t})$, where $A(s_{t})$ is the set of actions available in state $s_{t}$. The policy amounts to a conditional probability π(a|s) of the agent taking action if the current state is *s*. It is a mapping from state and action to the probability of taking an action. After that, the agent will receive a scalar reward $r_{t}$ and store the transition in the agent’s memory as experiences. The process continues until the agent reaches a terminal state. The agent seeks to learn a policy $\pi^{*}$ that maximizes the expected discounted return $R_{t}=\sum_{k=0}^{\infty }\gamma^{k}r_{t+k}$, accumulated reward with the discount factor γ∈(0,1] trades-off the importance of immediate and future rewards [19].

RL tasks that satisfy the Markov property can be described as Markov decision processes (MDPs), which are defined by a tuple (S,A,P,R,γ), where R is a reward function R(s,a) and P is a state transition probability $P(s_{t+1}|s_{t},a_{t})$. The Markov property indicates the future states are conditionally independent of the past given the present. So, in an RL task, the decisions and values are assumed to be a function only of the current state. Markov property can be defined as $p\left(s_{t+1}|s_{1},a_{1},\ldots ,s_{t},a_{t}\right)=p\left(s_{t+1}|s_{t},a_{t}\right)$, which means the future states are conditionally independent of the past given the present. RL task which satisfies Markov property can be described as MDPs, defined by the 5-tuple (S,A,P,R,γ), where R is reward function R(s,a) and P is state transition probability $P(s_{t+1}|s_{t},a_{t})$. In an episodic task, the state will reset after each episode of length, and the sequence of states, actions, and rewards in an episode constitute a trajectory or rollout of the policy [18].

### 3.1. Value Functions

Value functions are a core component of RL systems, which constructs a function approximator that estimates the long-term reward from any state. It estimates how good (expected return) it is for an agent to be in a given state (or given action in a given state) [44]. By this way, the function approximator exploits the structure in the state space to efficiently learn the value of observed states and generalize to the value of similar, unseen states [45]. A typical form of value function can be defined as:

(1) $$V^{\pi }\left(s\right)=E\left[R|s,\pi \right]=E\left[\sum_{k=0}^{\infty }\gamma^{k}r_{t+k}|s,\pi \right]$$

Normally we refer to $V^{\pi }\left(s\right)$ (1) as the state-value function, which measures the expected discounted return when starting in a state *s* and following a policy π. When actions follow by the optimal policy $\pi^{*}$, the state-value function can be optimal:

(2) $$V^{*}\left(s\right)=\max_{\pi }V^{\pi }\left(s\right)\;\forall s\in S$$

In addition to measuring the value of states, there is also an indicator for measuring the quality of actions’ selection, which is denoted as state-action-value or quality function $Q^{\pi }\left(s,a\right)$. It defines the value of choosing an action *a* from a given state *s* and thereafter following a policy π.

(3) $$Q^{\pi }\left(s,a\right)=E\left[R|s,a,\pi \right]=E\left[\sum_{k=0}^{\infty }\gamma^{k}r_{t+k}|s,a,\pi \right]$$

State-action-value is similar to the state value $V^{\pi }$ except the initial action *a* is provided, and the policy π is only followed from the succeeding state onwards. The optimal state-action-value function is denoted as:

(4) $$Q^{*}\left(s,a\right)=\max_{\pi }Q^{\pi }\left(s,a\right)\;\forall s\in S,\forall a\in A$$

$Q^{*}\left(s,a\right)$ gives the maximum state-action value for state *s* and action *a* achievable by any policy. This action-value function satisfies a recursive property, which is a fundamental property of value functions in the RL setting, and it expresses a relationship between the value of a state and its successor states:

(5) $$Q^{\pi }\left(s,a\right)=E_{s^{\prime }}\left[r+\gamma E_{a^{\prime }∼\pi \left(s^{\prime }\right)}\left[Q^{*}\left(s^{\prime },a^{\prime }\right)\right]|s,a,\pi \right]$$

Unlike producing absolute state-action values as with $Q^{\pi }$, an advantage function represents relative state-action values, which measures whether or not the action is better or worse than the policy’s default behavior [46]. Often, it is easier to learn that action yields higher reward than another, than it is to learn the actual return from taking one particular action [18]. Advantage function expresses a relative advantage of actions through this simple relationship:

(6) $$A^{\pi }\left(s,a\right)=Q^{\pi }\left(s,a\right)-V^{\pi }\left(s\right)$$

Many successful value-based RL algorithms [32,35,46] rely on the idea of advantage updates. In our approach, we also adopt the advantage value to measure the relative actions’ quality on each step.

### 3.2. Deep Q-Network

Deep reinforcement learning (DRL) applies deep neural nets for representing the value functions within reinforcement learning methods. DRL algorithms have attained superhuman performance in several challenging task domains to attribute to the powerful function approximation and representation learning properties of the DL. The DQN algorithm [47] achieves human-level performance on Atari series games from pixels input. It parameterizes the quality function *Q* with a neural network Q(s,a;θ) that approximates the *Q* values. Two main techniques of the DQN algorithm can learn value functions in a stable and robust way are using the target network and experience replay. At each iteration, the network’s parameters are updated by minimizing the following loss function:

(7) $$L_{i}\left(\theta_{i}\right)=E_{s,a,r,s^{\prime }}[{\left(y_{i}^{DQN}-Q\left(s,a;\theta_{i}\right)\right)}^{2}]$$

with

(8) $$y_{i}^{DQN}=r+\gamma \underset{a^{\prime }}{max}Q\left(s^{\prime },a^{\prime };\theta^{-}\right)$$

in which $\theta^{-}$ is the parameter for the target network. The first stabilizing method is fixing the target network’s parameters rather than calculating the TD error based on its own rapidly fluctuating estimates of the *Q*-values. The second one, experience replay, uses a buffer for storing a certain size of transitions $\left(s_{t},a_{t},s_{t+1},r_{t+1},\right)$ makes it possible for training off-policy and enhancing the efficiency of sampling data.

There is a series of improvements in the value-based RL setting after the DQN algorithm ignited this field. To reduce the overestimated *Q*-values in DQN, van Hasselt et al. [23] proposed the double DQN algorithm. Wang et al. [19] presented a dueling Q-network architecture to estimate state-value function V(s) and associated advantage function A(s,a) respectively. Tamar et al. [48] proposed a value iteration network that can effectively learn to plan, and it leads to better generalization in many RL tasks. Schaul et al. [24] developed the PER approach built on top of double DQN, it makes the experience replay process more efficient and effective than all transitions are replayed uniformly.

### 3.3. Dueling Network Architecture

**Figure 1:**
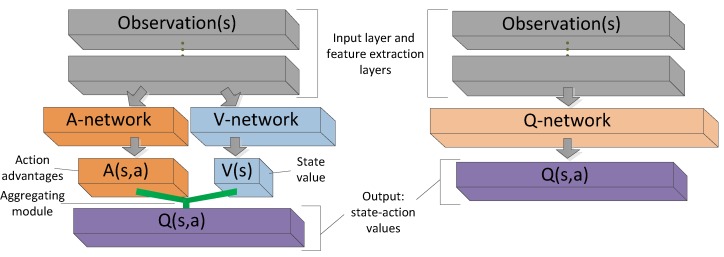
Dueling *Q*-network (**left**) and standard single-stream *Q*-network (**right**). Both networks have the same input and feature extraction module; the outputs are state-action values. The difference is that dueling network adopts two sequences (streams) networks to estimate state values and action advantages, and then combines them to indirectly generate the *Q*-values; The *Q*-network has a single sequence network and it directly produces *Q*-values’ estimation.

Unlike the standard single sequence *Q*-networks design (Figure 1 right), the dueling network structure (Figure 1 left) consists of two sequences (streams) of networks (A-network and V-network) which separately learn action-advantage function and state-value function. This construction decouples the value and advantage functions and combines the two streams to produce the estimate of the state-action value function with a special aggregating module (Figure 1 green module). The two streams share a common feature extraction layer (or lower layers). The deep *Q*-network focuses on estimating every state-action pairs’ value. However, the idea of dueling network is to estimate action-independent state function and action-dependent advantage function separately, because in RL environments, not all states are related to a specific action, there are many states independent of action, and under these states the agent does not need to change actions to adapt to the new states. Therefore, it is meaningless and inefficient to estimate such state-action pairs’ value. Dueling network firstly presented by Wang et al. [19] and through this change, the training efficiency has been greatly improved than the single-stream *Q* networks. The dueling network results in a new state of the art for tasks in the discrete action space according to Wang’s work. Shortly, the *Q*-values generated by dueling network are more advantageous to the performance improvement than deep *Q*-network in an RL task. In our approach, we adopt a dual-network design similar to dueling architecture to generate appropriate *Q*-values. In Section 4, we discuss the ADC network’s architecture and the aggregating method in detail.

### 3.4. Policy Gradient

The methods mentioned above indirectly learn the policy π(s) based on the estimate of the value functions. These value-based approaches are effective in handling problem in a discrete actions field. However, when dealing with a problem with a continuous action space such as physical control tasks, the value-based approaches cannot be straightforwardly applied, and it is difficult to ensure the results’ convergence since it relies on each actions’ *Q* value [49]. An obvious approach to implement value-based algorithms such as DQN to continuous domains is to discretize the action space to several fixed actions. However, it has many drawbacks and limitations such as throwing information (maybe essential) about the structure of the action domain [20].

There is no such worry in policy-based approaches since the policy network output agent’s actions without the estimation of the action-value function. They directly parameterize the control policy π(a|s;θ) and update the parameters θ [35] to optimize the cumulative reward, therefore, policy-based methods are more applicable to continuous control problem such as tasks of robotic controls [20,50,51,52,53] than the value-based methods. Policy gradient (PG) is an appealing policy-based algorithm which optimizes the parametric policy $\pi_{\theta }\left(a|s\right)=P\left[a|s;\theta \right]$ following the gradient $\nabla_{\theta }J\left(\pi_{\theta }\right)$ of its expectation of cumulative reward with respect to the policy parameters [54]. Policy-gradient methods are effective in high-dimensional or continuous action spaces, and can learn stochastic policies. In an RL task, the agent’s goal is to find parameter θ maximizes the objective function J(π). A typical performance objective to be considered is the average reward function: $J\left(\pi \right)=E\left[R|\pi_{\theta }\right]$. The policy-gradient theorem [33] provides the gradient of *J* with respect to the parameters θ of policy π:

(9) $$\begin{array}{c} \nabla_{\theta }J\left(\pi_{\theta }\right)=\int_{S}^{}\rho^{\pi }\int_{A}^{}\nabla_{\theta }\pi_{\theta }\left(a|s\right)Q^{\pi }\left(s,a\right)dads\\ \;\;\;\;=E_{s∼\rho^{\pi },a∼\pi^{\theta }}\left[\nabla_{\theta }log\pi^{\theta }\left(a|s\right)Q^{\pi }\left(s,a\right)\right] \end{array}$$

where the $\rho^{\pi }\left(s\right)$ is the state distribution. The unknown part, $Q^{\pi }\left(s,a\right)$ is normally estimated by using the actual returns $R_{t}=\sum_{k=0}^{\infty }\gamma^{k}r_{t+k}$ as an approximation for each $Q^{\pi }\left(s_{t},a_{t}\right)$ [33]. Based on this theorem, Silver et al. [49] proposed a deterministic policy-gradient (DPG) algorithm for estimating gradient and it is more efficient than the usual stochastic policy-gradient method. O’Donoghue et al. [36] referred to a new technique by combining PGQL and discussed the practical implementation of this technique in RL setting. In this paper, we consider the deterministic policies $a=\pi_{\theta }\left(s\right)$ because they significantly outperform their stochastic counterparts in continuous action spaces [49].

### 3.5. Actor-Critic Algorithm

Regular policy-gradient methods often exhibit slow convergence due to the large variances of the gradient estimates. The actor-critic methods attempt to reduce the variance by adopting a critic network to estimate the value of the current policy, which is then used to update the actor’s policy parameters in a direction of performance improvement [55]. The action-selection policy is known as the actor $\pi_{\theta }:S\to A$, which make decisions without the need for optimization procedures on a value function, mapping representation of the states to action-selection probabilities [33]. The value function is known as the critic $Q_{\phi }^{\pi }:S\times A\to R$, which estimates the expected return to reduce variance and accelerate learning [56], mapping states to expected cumulative future reward.

**Figure 2:**
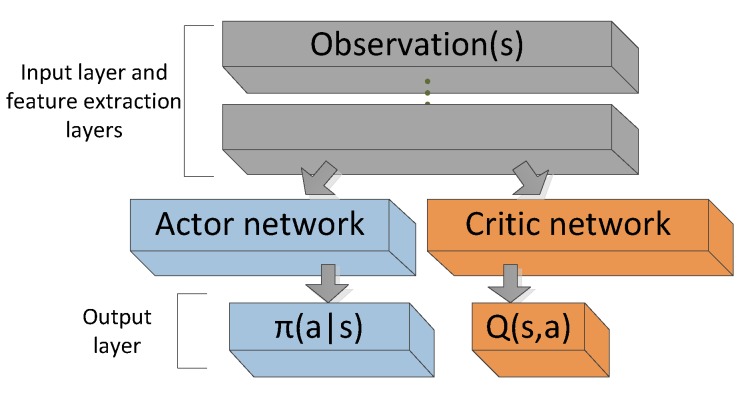
Actor-critic network.

Figure 2 shows an architecture design, the actor and critic are two separated networks share a common observation. At each step, the action selected by actor network is also an input factor to the critic network. In the process of policy improvement, the critic network estimates the state-action value of the current policy by DQN, then actor network updates its policy in a direction improves the *Q*-value. Compared with the previous pure policy-gradient methods, which do not have a value function, using a critic network to evaluate the current policy is more conducive to convergence and stability. The better the state-action value evaluation is, the lower the learning performance’s variance is. It is important and helpful to have a better policy evaluation in the critic network. Policy-gradient-based actor-critic algorithms are useful in many real-life applications because they can search for optimal policies using low-variance gradient estimates [56]. Lillicrap et al. [20] presented the DDPG algorithm, which combines the actor-critic approach with insights from DQN, to solve simulated physics tasks and it has been widely used in many robotic control tasks. It uses two neural networks; the actor network learns a deterministic policy and the critic network approximates the Q-function of the current policy [57].

## 4. Proposed Method

**Figure 3:**
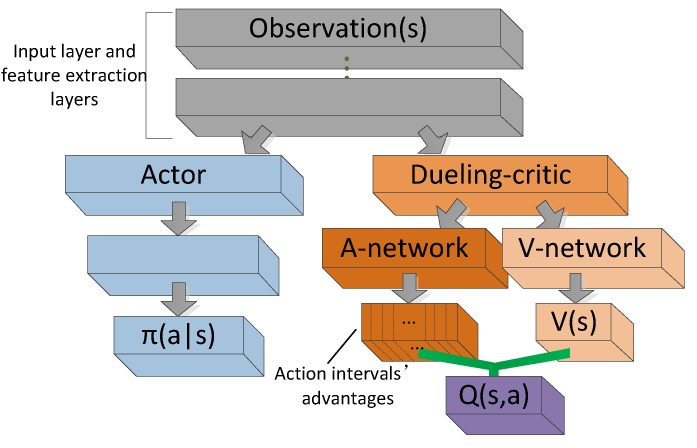
Actor-dueling-critic (ADC) networks architecture. It is based on actor-critic architecture. The actor network selects actions based on the policy-gradient method; The dueling-critic network applies dueling architecture to estimate state-action values. The ADC network has better *Q*-value estimator than the original actor-critic networks. The outputs of A-network are a list of action intervals’ advantages, the action space is uniformly divided. The aggregating module implements Equation (14) to combine the two streams.

In this work, we propose an approach for operating in continuous action space. We named our method as ADC network, which can find stable policies in continuous action spaces and it collects the benefits from the actor-critic network and dueling network. The main structure of the ADC network (Figure 3) is similar to the actor-critic network which consists of the two sequence networks. The actor network (left blue part in Figure 3) computes continuous actions with the DPG method. The dueling-critic network (right orange part in Figure 3) supplies the estimate of expected return as the performance’s knowledge for the actor. A difference from the actor-critic networks is the application of the dueling network (Figure 3 A-network and V-network) in the original critic branch. The dueling-critic network consists of two sequences (or streams) of fully connected layers which provide separate estimates of the state value V(s) and state-dependent advantage A(s,a). Then, the aggregating module combines the two streams to produce the estimate of state-action value *Q*. In the continuous action space, we cannot output the estimation of each possible action’s advantage value, so we add a new method to enable the dueling network to be used in continuous space, which was originally used in discrete action space. We manually divide the action space and estimate the advantage of the action interval in each state. Through this change, the agent could learn which action interval is good when facing a specific state and pick the action belong to this interval. The action-advantage value is a relative value and it measures the quality of the possible actions in one state. Meanwhile, it is a tiny amount close to zero and independent of the environment state and noise. Therefore, it can be seen as a fine-tuning factor to the *Q* value and improve the accuracy of *Q* value estimation.

The dueling-critic branch provides *Q* values to the actor, then the actor tends to know how good or bad the action taken is. So, an accurate *Q*-value estimation can lead to better performance for the actor-critic-based methods. In the traditional actor-critic methods, the critic applies a single sequence network and uses Q-learning updates to estimate state-action values, which force to build connections between states and actions. However, in practice, many states are independent of an action, which means in some states, the choice of action has no effect on what happens. Therefore, it is unnecessary to estimate each state-action pairs’ value. In our method, the dueling-critic decouples the action and state through this dual-network design. The value stream learns to pay attention to the state’s value; the advantage stream learns to pay attention to action interval’s advantage on a state, thus making the *Q* estimation more accurate by combining the two separate values. It also improves the computing efficiency. The original dueling network focuses on solving discrete actions’ RL problem. It cannot scale to continuous control tasks since it is a pure value-based method. However, the ADC method can cope with continuous action spaces since it has an actor network which is responsible for selecting actions based on policy. ADC combines the merit of dueling architecture and actor-critic frame. With an accurate state-action-value estimation, the actor-dueling-critic network can be more efficient in finding suitable policies than the classic actor-critic methods.

From the advantage Equation (6) we could get $Q^{\pi }\left(s,a\right)=V^{\pi }\left(s\right)+A^{\pi }\left(s,a\right)$. Then under the definition of advantage, we build an aggregating networks module:

(10) $$Q\left(s,a;\theta^{Q},\alpha ,\beta \right)=V\left(s;\theta^{Q},\beta \right)+A\left(s,a;\theta^{Q},\alpha \right)$$

where $\theta^{Q}$ denotes the parameters of the first layer in the dueling-critic branch, α and β are the network parameters of advantage and value streams respectively (A-network and V-network). The Q(s,a;θ,α,β) is the output of the dueling-critic network, and it is a parameterized estimate of true *Q*-function. Equation (10) lacks the identifiability since given *Q*, the *V* and *A* cannot be recovered uniquely. To migrate this issue, we force the *A* to have zero advantage at the chosen action:

(11) $$Q\left(s,a;\theta^{Q},\alpha ,\beta \right)=V\left(s;\theta^{Q},\beta \right)+(A\left(s,a;\theta^{Q},\alpha \right)-\max_{a^{\prime }\in \left|A\right|}A(s,a^{\prime };\theta^{Q},\alpha ))$$

Through this change, when $a=a^{*}=\arg\max_{a^{\prime }\in \left|A\right|}Q\left(s,a^{\prime };\theta^{Q},\alpha ,\beta \right)=\arg\max_{a^{\prime }\in \left|A\right|}A(s,a^{\prime };\theta^{Q},\alpha )$, the advantage equal to zero and then the *Q* equal to *V*. An alternative equation of aggregating module presented by Wang et al. [19] is:

(12) $$Q\left(s,a;\theta^{Q},\alpha ,\beta \right)=V\left(s;\theta^{Q},\beta \right)+\left(A\left(s,a;\theta^{Q},\alpha \right)-\frac{1}{\left|A\right|}\sum_{a^{\prime }}A(s,a^{\prime };\theta^{Q},\alpha )\right)$$

It replaces the max operator with the mean. Equation (12) increases the stability of the optimization because the advantages only need to change as the same pace of mean rather than compensate change to the optimal action’s advantage [19]. It also helps identifiability and does not change the relative rank of *A*. The original intention of advantage technique is to measure the relative value by comparing multiple actions under a state in discrete action spaces. While in this work, we focus on the continuous action space, so we uniformly partition the action space to *n* intervals (Figure 3) according to its experimental environment, and we use *z* denotes of the action interval. At each step, the A-network outputs the advantages of each action intervals ($z_{1},z_{2},\ldots ,z_{a},\ldots ,z_{n-1},z_{n}$), and we use the advantage value of interval ($z_{a}$) containing the action actor network adopted subtract the mean of all intervals’ advantage to calculate the step advantage. The definition of advantage’s value of the step when the agent takes an action *a* can be calculated with Equation (13):

(13) $$A\left(s,a;\theta^{Q},\alpha \right)=A\left(s,z_{a};\theta^{Q},\alpha \right)-\frac{1}{n}\sum_{z}A(s,z;\theta^{Q},\alpha )$$

Therefore, the equation of aggregating module of ADC network can be presented as (14):

(14) $$Q\left(s,a;\theta^{Q},\alpha ,\beta \right)=V\left(s;\theta^{Q},\beta \right)+A\left(s,a;\theta^{Q},\alpha \right)$$

In the actor network branch, we apply the off-policy DPG algorithm [49]. We parameterize the policy as $\mu (s|\theta^{\mu })$ which mapping states to a specific action (μ:S→A). The actor network adjust its parameters $\theta^{\mu }$ of the policy in the direction of the performance gradient $\nabla_{\theta^{\mu }}J$:

(15) $$\begin{array}{c} \nabla_{\theta^{\mu }}J\approx E_{s∼\rho }\left[\nabla_{\theta^{\mu }}Q\left(s,a|\theta^{Q}\right){|}_{a=\mu (s|\theta^{\mu })}\right]\\ \;\;\;\;\;\;\;=E_{s∼\rho }[\nabla_{a}Q\left(s,a|\theta^{Q}\right){|}_{s,a=\mu_{\left(s\right)}}\nabla_{\theta_{\mu }}\mu \left(s|\theta^{\mu }\right){|}_{s}] \end{array}$$

To ensure adequate exploration of action space, we execute an exploration policy by injecting noise into the output of action choice: $\mu^{\prime }\left(s\right)=\mu \left(s|\theta^{\mu }\right)+N$. Where the noise signal N randomly sampled from a Gaussian distribution. With this strategy, a noised action is taken with probability ϵ and the noise-free action is chosen with probability 1−ϵ. As the number of iterations increases, the ϵ slowly decreases from 1 to 0, at the same time, the actor network chooses noise-free action with increasing possibility from 0 to 1.

As the success of DQN algorithm, we use a neural network to learn in minibatches with a finite-sized cache R. Transitions were sampled from the environment according to the exploration policy. At each time steps, the actor and dueling-critic networks are updated by sampling a minibatch uniformly from the replay buffer R. Furthermore, for improving the stability of the training process we use the ’soft’ target updates in actor and dueling-critic networks rather than directly copy the target networks’ parameters. We set $Q^{\prime }\left(s,a|\theta^{Q^{\prime }}\right)$ and $\mu^{\prime }\left(s|\theta^{\mu^{\prime }}\right)$ as the target networks of dueling-critic and actor networks, respectively. The ’soft’ update means we update parameters slowly track the learned networks: $\theta^{\prime }\leftarrow \tau \theta +\left(1-\tau \right)\theta^{\prime }$ with τ≪1.

Figure 3 shows the basic architecture of our proposed network and Algorithm 1 provides the overall steps of our off-policy variant ADC algorithm.

**Algorithm 1** Actor-dueling-critic algorithm
1: **Initialize:**
 Initialize actor $\mu (s|\theta^{\mu })$ and dueling-critic $Q(s,a|\theta^{Q},\alpha ,\beta )$
 Initialize target actor $\mu^{\prime }$ with $\theta^{\mu^{\prime }}=\theta^{\mu }$ and target dueling-critic $Q^{\prime }$ with $\theta^{Q^{\prime }}=\theta^{Q},\alpha^{\prime }=\alpha ,\beta^{\prime }=\beta$
 Initialize replay memory R=∅, random process N.
 Uniformly separate the action space to *n* intervals ($Z=\left\{z_{1},z_{2},\ldots ,z_{n}\right\}$).
2: **for** episode=1 to M **do**
3:   Receive initial state $s_{1}$
4:   **for** t=1 to N **do**
5:    With probability ϵ select action $a_{t}=\mu \left(s_{t}|\theta^{\mu }\right)+N_{t}$, otherwise select $a_{t}=\mu \left(s_{t}|\theta^{\mu }\right)$
6:    Execute $a_{t}$ and observe reward $r_{t}$ and new state $s_{t+1}$
7:    Store transition $\left(s_{t},a_{t},r_{t},s_{t+1}\right)$ in *R*
8:    Sample a random minibatch of *N* transitions $\left(s_{i},a_{i},r_{i},s_{i+1}\right)$ from *R*
9:    Implement target actor $a_{i+1}^{\prime }=\mu^{\prime }\left(s_{i+1}|\theta^{\mu^{\prime }}\right)$
10:    Implement dueling-critic $Q_{i+1}^{\prime }=Q^{\prime }\left(s_{i+1},a_{i+1}^{\prime }|\theta^{Q^{\prime }},\alpha^{\prime },\beta^{\prime }\right)$ (Equation (14)) with $a_{i+1}^{\prime }\in z_{j}$
11:    Set $y_{i}=r_{i}+\gamma Q_{i+1}^{\prime }$ (set $y_{i}=r_{i}$ if $s_{t+1}$ is terminal)
12:    Update dueling-critic by minimizing the loss:         $L=\frac{1}{N}\sum_{i}{\left(y_{i}-Q\left(s_{i},a_{i}|\theta^{Q},\alpha ,\beta \right)\right)}^{2}$
13:    Update actor using the sampled PG:         $\nabla_{\theta^{\mu }}J\approx \frac{1}{N}\sum_{i}\nabla_{a}Q\left(s,a|\theta^{Q}\right){|}_{s=s_{i},a=\mu \left(s_{i}\right)}\nabla_{\theta^{\mu }}\mu \left(s|\theta^{\mu }\right){)|}_{s_{i}}$
14:    Soft update target networks of dueling-critic and actor (τ≪1):         $\begin{array}{cc} \theta^{\mu^{\prime }}\leftarrow \tau \theta^{\mu }+\left(1-\tau \right)\theta^{\mu^{\prime }} & \theta^{Q^{\prime }}\leftarrow \tau \theta^{Q}+\left(1-\tau \right)\theta^{Q^{\prime }} \end{array}$         $\begin{array}{cc} \alpha^{\prime }\leftarrow \tau \alpha +\left(1-\tau \right)\alpha^{\prime }\; & \;\beta^{\prime }\leftarrow \tau \beta +\left(1-\tau \right)\beta^{\prime } \end{array}$
15:   **end for**
16: **end for**


## 5. Experiments

We evaluated our approach on gym classic control environment and a navigation task. They are in continuous action domain. Experiments include non-noisy and noisy environments to explore the stability of our method.

### 5.1. Non-Noise Environments

#### 5.1.1. Gym Pendulum-v0

We apply off-policy ADC, DDPG, actor-critic and dueling networks on gym ’Pendulum-v0’ platform. This is a common continuous domain physical environment. We manually separate the continuous action space to 25 discrete actions for running the dueling network. It is a common method for applying most of value-based RL algorithms in continuous action space. The division of continuous action space based on experience, by choosing 25 discrete actions can lead to converging at a good speed for the dueling network. The agent’s goal is to try to keep the pendulum standing up, and when it stands up the reward is 0, otherwise, the rewards are negative value.

For the ADC network architecture, we applied a small fully connected neural network with one hidden layer of 30 neurons in actor and dueling-critic networks separately. In the following hidden layers of dueling-critic branch we use 100 neuron and 20 neurons for advantage and value streams, the 100 neurons of advantage denote of 100 action intervals of action space; In the DDPG and actor-critic networks, the actor branch is the same as that of ADC and the critic branch use 30 neurons in the hidden layer. Because both DDPG and ADC are based on the actor-critic network, it is reasonable to compare their results’ difference by making the main bodies of two networks the same. At the same time, this neural network can ensure that DDPG has good performance; In the dueling network, the first hidden layer use 30 neurons and the following advantage and value layers use 100 and 20 neurons. Additionally, we list the important hyper-parameters in Table 1. The vanilla actor-critic approach we used without buffer and we did not apply soft replacement in dueling and actor-critic approaches.

**Figure 4:**
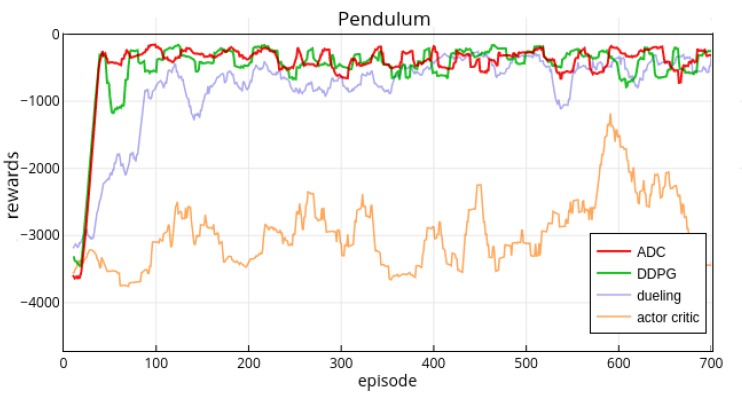
ADC, DDPG, dueling and actor-critic’s performance in the gym Pendulum-v0 environment. The x-axis represents the episodes and y-axis represents the cumulative rewards per episode. Table 1 lists important hyper-parameters.

**Figure 5:**
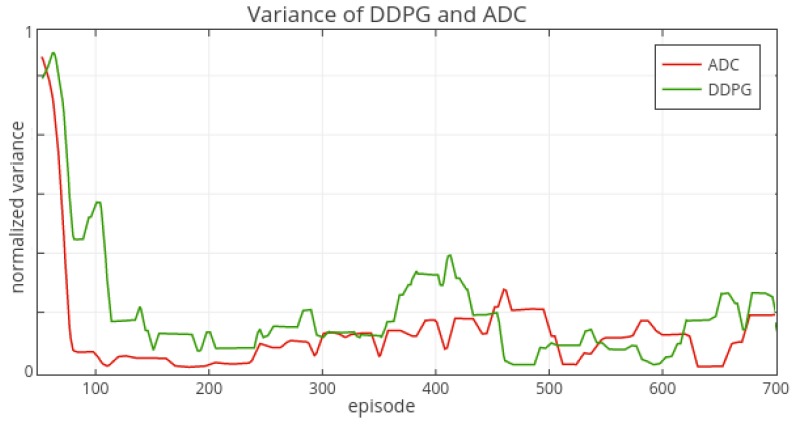
The variance of DDPG and ADC’s rewards.

As the Figure 4 shows, the vanilla actor-critic method performs poorly and fluctuates violently. It is hard to learn a good policy for vanilla actor-critic method without other technique such as experience replay; The dueling approach learns policy at a very slow speed and it can achieve good performance after the 200th episode but still behave unstable before the 400th episode; The ADC method can overcome the shortcomings of actor-critic and it learns a stable policy quickly. From beginning to the 50th episode the ADC and DDPG both achieve a good level and then ADC behaves more stable than DDPG (from the 50th episode to 200th episode). After the 200th episode, the performance of ADC and DDPG are at the same level. For comparing the stability of these two methods, we plot the variance Figure 5 of these two methods.

The variance of ADC is significantly lower than that of DDPG in the initial stage from the beginning to the 450th episode. After the 450th episode the DDPG’s variance tends to decrease, and then both variances stay stable. In the initial part (50th–300th episode), ADC has higher rewards than that of DDPG (Figure 4), and the rewards’ variance of ADC is lower than that of DDPG (Figure 5). Therefore, in this task, the ADC approach can learn better policy with high performance (rewards) and stability than the DDPG method. Obviously, ADC’s learning ability is also better than that of vanilla actor-critic and dueling approaches.

#### 5.1.2. Navigation Task

**Figure 6:**
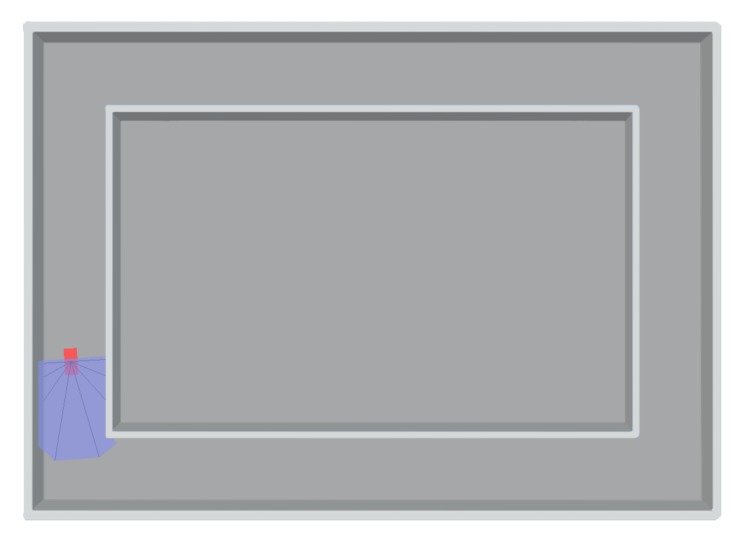
Obstacle avoidance task. The agent measures distance from obstacle with 8 sonars. The purpose is trying to go as far as possible without any collision.

In this navigation task, we implement ADC and DDPG methods in an obstacle avoidance task to test the long-term training performance. This is an obstacle avoidance task (Figure 6) in a continuous action space. The main goal of the agent (robot) is trying to go as far as possible and avoid the obstacles (wall). The agent has 8 sonars they measure the distances in 8 different directions, and it is given a little positive reward (0.001) for moving on each step without collision. Meanwhile, it can be punished with a negative reward (−1) for hitting obstacles. Every time the robot hit a wall, it will restart from the starting point. We set 500 steps per episode and 3500 episodes in total. The action is the steering angle range from $\left[-6^{\circ },+6^{\circ }\right]$ at each step. Based on the distance information of sensors and the rewards value, the robot can learn the correct way to avoid collisions. Through rewards drive, the robot knows what action can be avoided punishment. In this environment, the information received by the robot’s sonars is the state, which are distances from the obstacles in 8 directions. The steering operation is the action, these values make up a standard Markov process. The trained model is more flexible and applied to a variety of scenarios. This environment can properly simulate the operation of the real laser robot.

**Figure 7:**
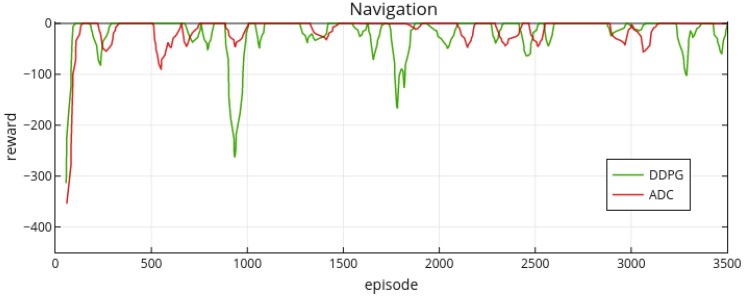
ADC and DDPG’s performance on obstacle avoidance task. The x-axis presents the episodes and y-axis presents the cumulative rewards per episode. The whole process has 3500 episodes and each episode has 500 steps.

The actor branches in ADC and DDPG architectures have two hidden layers with 100 neurons in the first hidden layer and 20 neurons in the second hidden layer. The critic branch of DDPG has 100 neurons and 20 neurons in the first and second hidden layers separately. The dueling-critic branch of ADC has 100 neurons in the first hidden layer, 100 neuron (advantage stream) and 20 neurons (value stream) in the second hidden layers separately. We list the important hyper-parameters in Table 2. The experimental results are shown in Figure 7.

**Figure 8:**
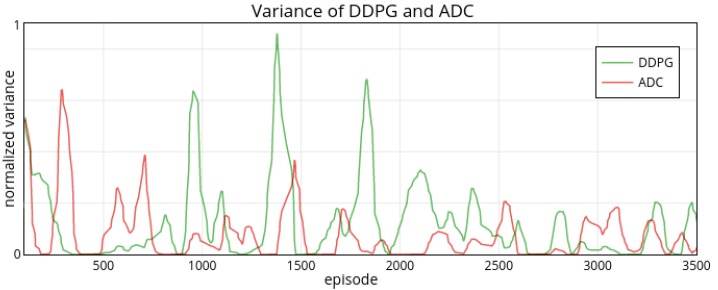
Variance of ADC and DDPG’s rewards.

From the overall performance, both approaches can achieve a similar performance level in this task, and they can quickly adapt to the environment within the first 100 episodes. However, from the stability, ADC is more stable than DDPG, especially in the 900th episode, 1800th episode and 3300th episode. For intuitively comparing the stability of these two methods’ rewards, we also plot the variance figure (Figure 8).

More intuitively, the variance of ADC’s rewards shows a significant downward trend, and it is less than DDPG’s variance at most of the episodes. The variance of the DDPG is particularly large at the 900th, the 1350th, and the 1800th episode. In contrast, ADC behaves more stable than DDPG in the whole process. After the 2500th episode, DDPG’s variance becomes less violently and maintain the same level as the ADC. To sum up, the ADC approach can tackle this continuous control task, and it can learn a more stable policy than DDPG method during long-term training.

### 5.2. Noise Environments

In this section, we do experiments in environments with noise input to test the training stability. We omitted the results of deep Q-learning, dueling network and actor-critic algorithms because they perform poorly. We mix random noise to each channel of the environment’s state as interference sources. The noise random samples from a uniform distribution over [−0.1,+0.1] (mean = 0, standard deviation = 0.0578). The introduced noise can reduce the learning efficiency of a model and cause a certain degree of instability, the convergence rate decreases in the first 200 episodes. The real robots also face environmental noise and affect the training process. Moreover, the state value mixed with noise affects the agent’s judgment and policy. Our method learns the policy based on state value and action advantage.

**Figure 9:**
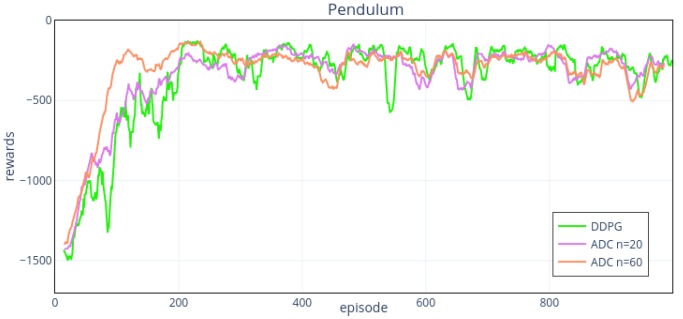
ADC and DDPG’s performance on Pendulum-v0 environment with noise input. The action intervals of ADC network are 20, 60, respectively. When *n* is 20, ADC has a limited promotion compared with DDPG. When *n* is 60, the convergence speed and stability have been greatly improved.

**Figure 10:**
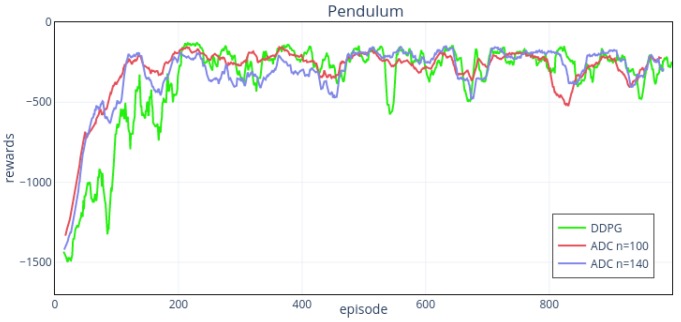
The action intervals of ADC network are 100, 140, respectively. The performance of the two groups are similar, both better than DDPG. The stability of 100 intervals is slightly better than 140.

We use the hyper-parameters in Table 3 and the same network structure as noise environment to operate in Pendulum-v0 environment noise input. To explore the effect of the number of action’s intervals on the outcome, we set four groups of intervals. Figure 9 and Figure 10 show the results.

From the results, the training effects are affected by noise disturbance, which is reflected in the slower learning rate and worse stability. Meanwhile, the performance of ADC method is better than that of DDPG in convergence speed and stability. When n=60,100,140, the training results can convergence at around 120th episode, while for DDPG, it is over the 200th episode. From Figure 9 shows, when n=20, the performance of ADC is slightly better than that of DDPG. While n=60, performance improvement is more obvious. When n=100,140 (Figure 10), the overall performance of the two is similar, and the stability of 100 intervals is slightly better. When n=100,140, they show a higher upward trend in the first 50 episodes than that of 20, 60 action intervals, but the upward trends become slower after the 50th episode.

## 6. Discussion

The combination of dueling architecture and actor-critic network allows our approach to use action advantages as an auxiliary value to *Q*-value estimations and hence helps the policy select a correct action in continuous action domain. As the non-noise experiment shows, our approach overcomes the shortcoming of actor-critic networks, which cannot learn a good policy, and the performance is significantly unstable. Meanwhile, the dueling network has a low learning rate in the continuous control task. The DDPG is a successful actor-critic-based method, it has good results in continuous control tasks. The ADC method can achieve better results with slightly higher stability. From the navigation task, it demonstrated that the ADC approach can attain long-term higher stability than DDPG in a non-noise environment. Furthermore, the ADC’s average reward for the whole period is also higher than that of DDPG. Meanwhile, the navigation task also proves the feasibility of our method in the field of real-world navigation. We directly applied the trained model to unseen simulator environments by changing the path and width, and the agent can avoid obstacles perfectly without any collision. It shows that the trained model has generalization ability. To further explore the performance of our method in the noise environment, we designed the second experiment. Meanwhile, the effects of different action intervals on the overall performance were researched. The experimental results show that ADC is more insensitive to the environment’s noise than DDPG; even the noise makes the performance of two fluctuate a little. From the exploration of different action intervals, the preliminary conclusion is that with a small number such as n=20, its improvement is not very obvious compared to DDPG, but when n=60,100, the overall effects are much better. If it further increases, such as n=140, the effect is not obviously improved. In addition, it increases the training time and computational resource. The specific impact of the action interval’s number needs further study. Overall, ADC and DDPG work well in continuous action spaces. In the noise environment, the learning efficiency and stability of ADC are better than that of DDPG.

## 7. Conclusions

This paper introduces a novel ADC approach for solving the obstacle avoidance task of sensor-based robots. These are continuous control problems. The ADC is based on the actor-critic network and it is more efficient than the original vanilla actor-critic method. Continuous control ability is a fundamental requirement for autonomous robots that interact with the real environment. We used the navigation scenario to test the performance of the ADC algorithm in the obstacle avoidance task. From the results, the obstacle avoidance problem in sensor-based robots can be well solved by using the ADC algorithm. To improve its training stability, we used a series of techniques such as experience replay, target network, soft update, ϵ-greedy etc. in its algorithm. The applications of these techniques make the learning process more stable and improve the sampling use rate in the replay buffer. In addition, since the traditional method of state-action estimation hinders the performance improvement of actor-critic-based algorithms, we introduce a dueling-critic network which decouples the states and actions and estimates state value and action interval advantage separately. By aggregating the two values—dueling-critic output the state-action values—then the actor network updates its parameters according to the *Q*-value. The dueling structure can improve the accuracy of *Q*-value estimation in noise environment by using advantage technique. Through the combination of the dueling and actor-critic network, the ADC can work well and be stable in a noise environment. We conduct experiments to examine the algorithm and compare it with other methods, a vanilla actor-critic network method, dueling network method, and DDPG method. In the gym Pendulum-v0 experiment, our approach can quickly adapt to the environment and show high efficiency and stability in dealing with continuous control problems. In the navigation environment, the results show our method can solve the obstacle avoidance problem and its training performance is stable and reliable Furthermore, we designed a noise environment to compare the training efficiency of ADC and DDPG. The superiority of ADC in the noise environment is more obvious. It indicates that our approach has made progress on training efficiency.

There are some problems we plan to address in future work. First, the stability and efficiency of the ADC network need further investigation, especially in the face of more complex problems and application scenarios. Second, the influence of interval advantage on performance needs to be further explored. Third, in dealing with the action interval advantage, we need to explore how to reasonably divide the action space and how to divide action space in a complex environment, such as adaptively dividing the action space. Fourth, the method will be transferred to a real laser robot to test performance in obstacle avoidance tasks.

## Figures and Tables

**Table 1:** Hyper-parameters.

Hyper-Parameter	ADC	DDPG	Actor-Critc	Dueling
Discount factor γ	0.9	0.9	0.9	0.9
Buffer size *R*	5000	5000	N/A	5000
Batch size	32	32	N/A	32
Learning rate α	a:0.001 c:0.002	a:0.001 c:0.002	a:0.001 c:0.002	0.001
Soft replacement τ	0.01	0.01	N/A	N/A

**Table 2:** Hyper-parameters.

Hyper-Parameter	ADC	DDPG
Discount factor γ	0.9	0.9
Buffer size *R*	5000	5000
Batch size	16	16
Learning rate α	a:1e−4 c:2e−4	a:1e−4 c:2e−4
Soft replacement τ	0.01	0.01

**Table 3:** Hyper-parameters.

Hyper-Parameter	ADC	DDPG
Discount factor γ	0.9	0.9
Buffer size *R*	5000	5000
Batch size	32	32
Learning rate α	a:1e−4 c:2e−4	a:1e−4 c:2e−4
Soft replacement τ	0.01	0.01
